# Long non‐coding RNA GAPLINC promotes angiogenesis by regulating miR‐211 under hypoxia in human umbilical vein endothelial cells

**DOI:** 10.1111/jcmm.14678

**Published:** 2019-10-07

**Authors:** Yangyan He, Ziheng Wu, Chenyang Qiu, Xiaohui Wang, Yilang Xiang, Tian Lu, Yunjun He, Tao Shang, Qianqian Zhu, Xun Wang, Qinglong Zeng, Hongkun Zhang, Donglin Li

**Affiliations:** ^1^ Department of Vascular Surgery The First Affiliated Hospital, College of Medicine, Zhejiang University Hang Zhou China

**Keywords:** angiogenesis, Bcl2, critical limb ischaemia, GAPLINC, hypoxia, long non‐coding RNA, miR‐211

## Abstract

In this study, we investigated the role of a long non‐coding RNA GAPLINC in angiogenesis using human umbilical vein endothelial cells (HUVEC). We found that hypoxia and hypoxia‐inducible factor 1α (HIF‐1α) increased the expression of GAPLINC in HUVEC cells. Moreover, GAPLINC overexpression down‐regulated miR‐211 and up‐regulated Bcl2 protein expression. Further rescue experiments confirmed that hypoxia directly increased GAPLINC expression. GAPLINC overexpression also increased cell migration and vessel formation which promoted angiogenesis, and these changes were attributed to the increased expression of vascular endothelial growth factor receptors (VEGFR) and delta‐like canonical notch ligand 4 (DLL4) receptors. Finally, we demonstrated that GAPLINC promotes vessel formation and migration by regulating MAPK and NF‐kB signalling pathways. Taken together, these findings comprehensively demonstrate that overexpression of GAPLINC increases HUVEC cells angiogenesis under hypoxia condition suggesting that GAPLINC can be a potential target for critical limb ischaemia (CLI) treatment.

## 1 INTRODUCTION

Critical limb ischaemia (CLI) is a clinical syndrome of ischaemia pain, rest pain, ischaemic ulceration and gangrene with a high risk of limb loss, cardiovascular disease and major amputation. It is considered to be the most fatal complications of peripheral artery disease (PAD).^1^ Diabetes is among the high‐risk factors of PAD.^2^ Currently, some of the treatment of CLI including vascular reconstruction, relieve pain and wound healing aimed at preventing limb amputation. However, many CLI patients do not respond to classical treatments and often require primary amputation.^3^, ^4^, ^5^ For these patients, a novel concept termed as “therapeutic angiogenesis” aimed at increasing local angiogenesis has been proposed as an effective treatment. Several trials have indicated that gene therapy targeted at factors such as vascular endothelial growth factor (VEGF) and hepatocyte growth factor (HGF) can potentially increase local angiogenesis.^6^, ^7^, ^8^ Recently, a US phase II and a Japanese phase III clinical trial on HGF gene therapy for CLI demonstrated that HGF gene therapy significantly improved the primary end‐points and increased partial oxygen pressure.^6^, ^7^ However, the optimal dose and duration of application of either single or multiple growth factors for gene therapy have not been determined for CLI patients.

Current RNA sequencing studies have indicated that the proportion of the genome responsible for protein coding is approximately 1.5%, while the majority of the genome is non‐coding RNA (ncRNA). Among the types of ncRNAs, long non‐coding RNA (lncRNA) is transcribed RNA molecules >200 nt in length.^9^, ^10^ Accumulating evidence suggests that lncRNAs play important roles in the different biological process such as cell growth and differentiation, apoptosis and immune responses. Indeed, deregulation of lncRNA has been associated with different human diseases, including cancer, tumorigenesis and neurological diseases.^11^, ^12^ LncRNAs are predominantly found in the cytoplasm and nucleus suggesting that lncRNAs regulate cell functions in both locations. Indeed, studies have shown that lncRNAs can control gene expression by epigenetic regulation and also influencing transcription factors.^12^ Several aspects of lncRNA can be exploited for therapeutic purposes because of two major advantages. Firstly, lncRNA can manipulate target proteins previously considered to be undruggable. Secondly, they can be targeted with higher specificity than other small molecules.^13^ For instance, the recently identified GAPLINC is a 924‐bp lncRNA, that is involved in different types of cancer.^14^, ^15^, ^16^, ^17^ In gastric cancer and colorectal cancer, GAPLINC competes with CD44 for miR‐211, and higher expression of GAPLINC was found to be correlated with large tumour size.^14^ High expression of GAPLINC increases cell migration and vessel formation. These observations suggested that GAPLINC can increase local angiogenesis by modulating several genes. Therefore, we hypothesized that GAPLINC may possess therapeutic potential for CLI. In this study, we aimed at understanding the molecular mechanism of angiogenesis under hypoxia condition and aimed to identify novel therapies for CLI based on lncRNAs.

## 2 MATERIAL AND METHODS

### 2.1 Ethical approval of the study protocol

All research involving human participants was approved by the Institutional Review Board (IRB) of the Ethics Committee of Zhejiang University. Informed consent was obtained from each patient. The study protocol was approved by the Institutional Animal Care and Use Committee of Zhejiang University. The study was conducted following international guidelines for animal experimentation.

### 2.2 Cell culture

HUVEC cells were purchased from ATCC cell bank. The cells were cultured in F‐12K medium and maintained at 37°C in the presence of 5% CO_2_ incubator.

### 2.3 qPCR

Total RNAs were isolated from cell lysate using the TRIzol Reagent (Aidlab). The concentration and quality of RNA were checked by Nono‐100. The cDNA was generated using Hiscript Reverse transcriptase (VAZYME) by random primers. GAPDH was used as internal control. The qPCR reaction was conducted using the SYBR Green at 50°C for 2 minutes, 95°C for 10 minutes, followed by 40 cycles of 95°C for 30 seconds and 60°C for 30 seconds. The relative expression of each mRNA or miRNA was calculated using the comparative Ct method. The experiments were performed using an ABI QuantStudio6 (Applied Biosystems). All the primers used are listed in Table S1.

### 2.4 Construction of adenovirus expression vectors

We used adenovirus vectors for both overexpression and silencing of GAPLINC. The sequence of GALPLINC was derived from pUC57‐GAPLINC, and the sequence can be accessed on the NCBI references file (NR_110429.1). For overexpression vectors, the GAPLINC fragment was digested by BamHI and Xhol from pUC57‐GAPLINC plasmid which was then ligated into the digested pBF‐adshuttle plasmid as a new plasmid, named as pBF‐adshuttle‐GAPLINC. GAPLINC RNAi sequence (CCTGAAATAATGAACTCCT) was ligated into pG1.1 plasmid to form a new plasmid (pG1.1‐homoGAPLIN‐56‐1). Both overexpression and silencing vectors were recombined with pAd/PL‐DEST to form adenovirus vectors. Both vectors were transfected into HEK293 cells and then harvested at 72 hours after transfection. The virus titre for both plasmids was 6 × 109 and 2 × 109 TU/mL, respectively.

### 2.5 Protein extraction and western blot analysis

Cells were washed thrice with cold PBS buffer (0.01 mol/L, pH = 7.2) and then mixed with 400 µL of 1 mmol/L PMSF lysis buffer for 30 minutes on ice. The proteins were obtained by centrifugation at 3800 *g* for 5 minutes. The protein concentration was achieved by the BCA method. Protein samples were subjected to SDS‐PAGE and then transferred to PVDF membrane. The primary antibodies raised against GAPDH (1:1000, Goodhere), Bcl2 (1:1000, Proteintech), VEGFR (1:2000, Boster) and DLL4 (1:300, Proteintech) were incubated with the membrane overnight. Then the membranes were incubated with secondary antibodies (1:5000) at room temperature for 2 hours. Finally, the data were analysed by BandScan.

### 2.6 Cell migration assay

The 24 transwell plates (BD Biosciences) were used for cell migration assay. The 200 µL of the cell suspension was added into each well and incubated for overnight. The cells that had migrated through the membrane were fixed with 70% ice ethanol for 1 hour and then stained with crystal violet for 20 minutes. The sample was washed with PBS and visualized by microscopy. The mean number of migrated cells was recorded in five randomly selected fields at 200× magnification in each membrane.

### 2.7 Vessel formation assay

The cells subjected to different conditions were seeded in 6‐well plates. The Matrigel (Corning) was dissolved, and the tips/24‐well plate were pre‐chilled at 4°C overnight. A volume of 100 µL of Matrigel was added to the 24‐well plate and then centrifuged to remove bubbles. After centrifugation, the plates were pre‐warmed at 37°C for 1 hour and 15 × 10^4^ cells were added into each well. Thereafter, the plates were cultured for another 8 hours and the samples were washed with PBS and visualized by microscopy. The vessel formation was recorded in five randomly selected fields at 100× magnification in each well.

### 2.8 Rescue experiments

Subsequently, the rescue experiment of GAPLINC was carried out in HUVEC cells. Cells were treated under hypoxia condition before transfected with GAPLINC siRNA. The expression level of lncRNA GAPLINC and miR‐211 at 24 hours was assessed by qPCR assay, and the protein expression of Bcl‐2 was determined by Western blot. The cell migration and vessel formation assays were performed as described above.

### 2.9 Transcriptome analysis by RNA‐seq

The arteries tissue from three patients and three healthy individuals were lysed, and total RNA was extracted using total RNA isolation kit (Qiagen). The concentration of RNA was measured by spectrometer K5500, and the RNA integrity was checked by RNA 6000 Nano assay (Agilent) after removing the rRNA using Ribo‐Zero Gold kits. The library was prepared by NEB next Ultra directional RNA library prep Kit. The library was sequenced by HiSeq 2500 instrument (Illumina) with 125 bp pairing end. The quantification of transcript abundance was conducted using RSEM software (v1.2.22) supported by the STAR aligner software (STAR_2.4.2a). The DEseq was used for differentially expressed genes (DEGs). Diseases and function annotation were analysed by Ingenuity Pathway Analysis (IPA v48207413), which can analyse the gene expression patterns using a build‐in scientific literature‐based database,^18^ and gene set enrichment analysis was performed by WebGestalt online tools, which is well‐established and well‐maintained gene set enrichment analysis toolkit (2019version).^19^

### 2.10 Statistical analysis

Data are presented as mean ± SD. All statistical analyses were performed using analysis of a Turkey multiple comparison test on GraphPad Prism 7. Spearman correlation test was applied for correlation assessment. A *P*‐value < .05 was considered statistically significant.

## 3 RESULTS

### 3.1 Transcriptional profiling of arteries tissue gene expression in CLI patients

**Figure 1:**
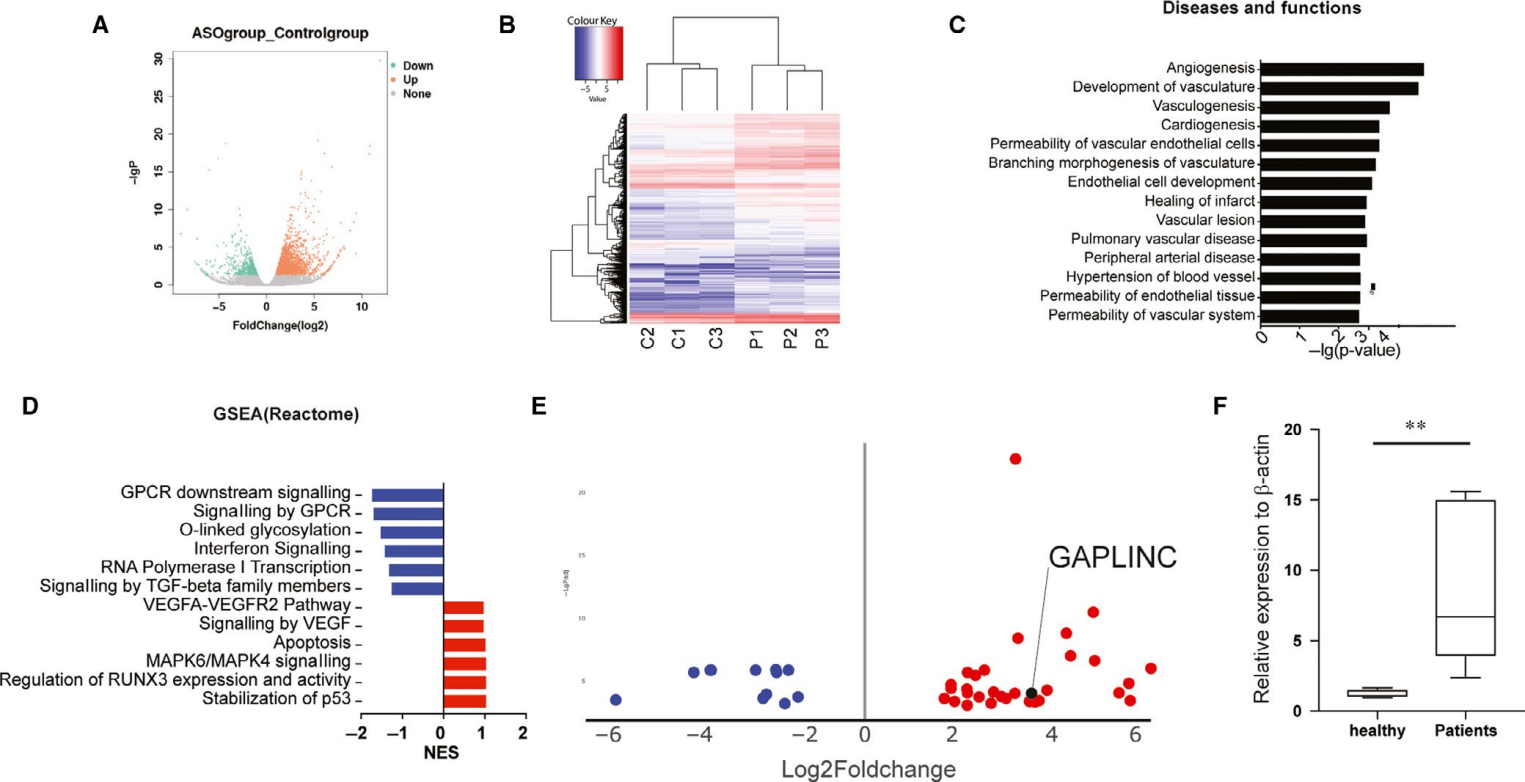
Profound changes in the global transcriptional landscape. A, Volcano plot displaying genes detected by RNA‐Seq. Green dots represent genes which show down‐regulated genes; red dots indicate up‐regulated genes. Nominal *P* < .05. B, Hierarchical clustering and heatmap of genes that are differentially expressed (DEGs) between CLI patients and healthy donors. C, Functional annotations of differentially expressed genes, as determined using Ingenuity Pathway Analysis. The value is lg10 *P*‐value. D, Gene set enrichment analysis was analysed by WebGestalt. The Reactome pathway results were shown here. The value is a normalized enrichment score determined by the programme. E, Volcano plot displaying lncRNA detected by RNA‐Seq. Blue dots represent genes which show down‐regulated genes; red dots indicate up‐regulated genes. Nominal *P* < .05. F, The expression level of GAPLINC was validated by CLI patient sample. **<.01

To obtain a global perspective of transcriptional changes arteries tissue from critical limb ischaemia patients, therefore, we conducted bulk RNA‐Seq analysis of transcriptional profiling experiments to characterize gene expression profiling in the three CLI patients compared with three healthy donors (Table S2). We observed that on a global transcriptional level, gene expression signatures are different from each other with a total of n = 1978 transcripts showing differential gene expression (DEGs, nominal *P* < .05) shown in Figure 1A and B. Notably, functional annotations of DEGs mainly referred to vascular disease and functions, including such as angiogenesis, vasculogenesis by Ingenuity Pathway Analysis (IPA) software (Figure 1C). We also performed Gene Set Enrichment Analysis (GSEA), and the results showed that GPCR (G‐protein‐coupled receptors) pathway and TGF‐β pathway were down‐regulated in CLI patient. In contrast, VEGF (vascular endothelial growth factor) pathway mainly involving in vascular functions and diseases pathways was up‐regulated (Figure 1D). We next mapped all possible lncRNAs from our data set, and 43 differentially expressed lncRNAs were observed (nominal *P* < .05). We noticed that more than half of the lncRNA was up‐regulated in CLI patients including well‐known lncRNA GAPLINC (Figure 1E), and this up‐regulation of GAPLINC also confirmed by qPCR (Figure 1F). Given the known roles of GAPLINC lncRNA in angiogenesis and the functions of the GAPLINC in the context of hypoxia condition, we hypothesized that GAPLINC lncRNA might be a good a target for CLI diseases gene therapy.

### 3.2 GAPLINC are up‐regulated in HUVEC under low‐level oxygen conditions

**Figure 2:**
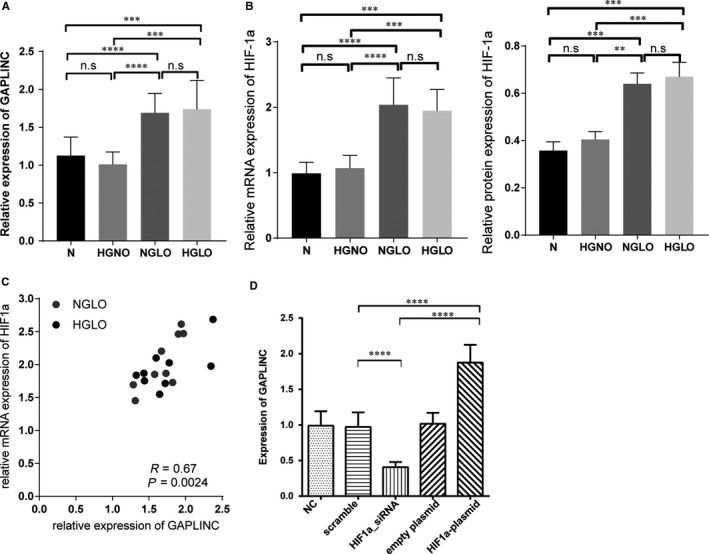
Increasing expression of GAPLINC under hypoxia condition. A, Relative expression of GAPLINC under different conditions measured by qPCR. B, Relative expression of HIF‐1α under different conditions measured by qPCR and at the protein level. C, The correlation between protein level of HIF‐1a and GAPLINC level. The Spearman correlation was used in this analysis. D, Relative expression of GAPLINC under HIF‐1a knockdown and overexpression condition. N: normal condition; HSNO: high sugar normal oxygen; NSLO: normal sugar lower oxygen level; HSLO: high sugar low oxygen condition. The results are presented as mean ± SD for three independent experiments. Each experiment performed in triplicate. *: *P* < .05, **: *P* < .01, ***: *P* < .001, ****: *P* < .0001

To develop the in vitro model using HUVEC cell line, we first checked whether the cell line expressed the target molecule. The qPCR and Western blotting experiments showed that the HUVEC cell line expresses GAPLINC and miR‐211. We next subjected HUVEC cells to different conditions to mimic the glucose and oxygen level in the diabetes condition. Strikingly, we found that the expression of GAPLINC was significantly increased under low oxygen condition regardless of glucose concentration (Figure 2A). Previous studies showed that increased expression of hypoxia‐inducible factors (HIFs) responded to low oxygen condition.^18^ Moreover, recent studies on tumour cell lines also found that the expression level of HIF‐1α was positively correlated with GAPLINC expression.^18^ Hence, we determined the HIF‐1α expression level at the different conditions at both mRNA and protein levels in HUEVC cells. The results indicated that HIF‐1α was highly expressed in low oxygen condition (Figure 2B). A significant positive correlation between GAPLINC and HIF‐1α expression (*R* = 0.67, *P* < .01, Figure 2C) suggesting that hypoxia condition modulates GAPLINC expression. To validate the correlation between GALPINC and HIF‐1α, we performed HIF‐1α knockdown and overexpression experiments. The results demonstrated that changes in GAPLINC expression level were accompanied by HIF‐1a, suggesting that HIF‐1a is the upstream partner of GALPLINC (Figure 2D).

### 3.3 GAPLINC modulates the expression level of Bcl‐2

**Figure 3:**
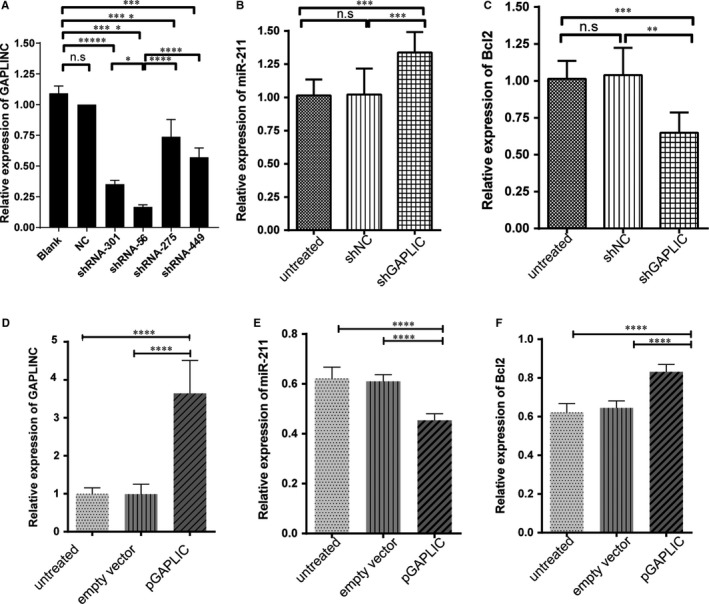
Expression of miR‐211 and Bcl‐2 in GAPLINC overexpression and knockdown condition. A, Different shRNA silencing abilities were tested for GAPLINC. B, Relative expression of miR‐211 when knowdown GAPLINC expression. C, Relative expression of Bcl‐2 when knowdown GAPLINC expression. D, Overexpression of GAPLINC in cell line by transfection. E, Relative expression of miR‐211 when overexpressing GAPLINC expression. F, Relative expression of miR‐211 when overexpressing GAPLINC expression. The results are presented as mean ± SD for three independent experiments. Each experiment performed in triplicate. *: *P* < .05, **: *P* < .01, ***: *P* < .001, ****: *P* < .0001

Our previous study showed that overexpression of Bcl‐2 can rescue the functions of HUEVC cell during high glucose and low oxygen conditions.^20^ However, the upstream regulator of Bcl‐2 was not clear under this context. Several results from cancer studies have been shown that GAPLNC can modulate the expression of Bcl‐2 by down‐regulating miR‐211.^14^, ^21^ Here, we tested this hypothesis by either silencing or overexpressing GAPLINC in HUEVC cells. Different shRNAs were designed according to GAPLINC sequence, and the silencing efficacy was checked by qPCR. Finally, shRNA‐56 displayed the best ability to inhibit the expression of GAPLINC and hence was used in the subsequent study (Figure 3A). When shRNA was transfected to silence GAPLINC expression, we found that the expression level of miR‐211 was significantly up‐regulated in shRNA knockdown cells compared with negative controls. In contrast, the expression level of Bcl‐2 was significantly down‐regulated (Figure 3B). Besides, the miR‐211 expression level was negatively correlated with Bcl‐2 expression implying that GAPLINC modulates the expression of Bcl‐2 through the miR‐211 pathway (Figure 3C). To further confirm this finding, we first overexpressed GAPLINC expression plasmid in HUVEC cells (Figure 3D). Overexpressing GAPLINC significantly down‐regulated the expression level of miR‐211, whereas it significantly up‐regulated Bcl‐2 expression corresponding to GAPLINC silencing results (Figure 3E and F). Taken together, these results suggested that GAPLINC serves as a molecular decoy for miR‐211 to modulate the expression of Bcl‐2.

### 3.4 Enhanced GAPLINC expression improves Migratory capacity

**Figure 4:**
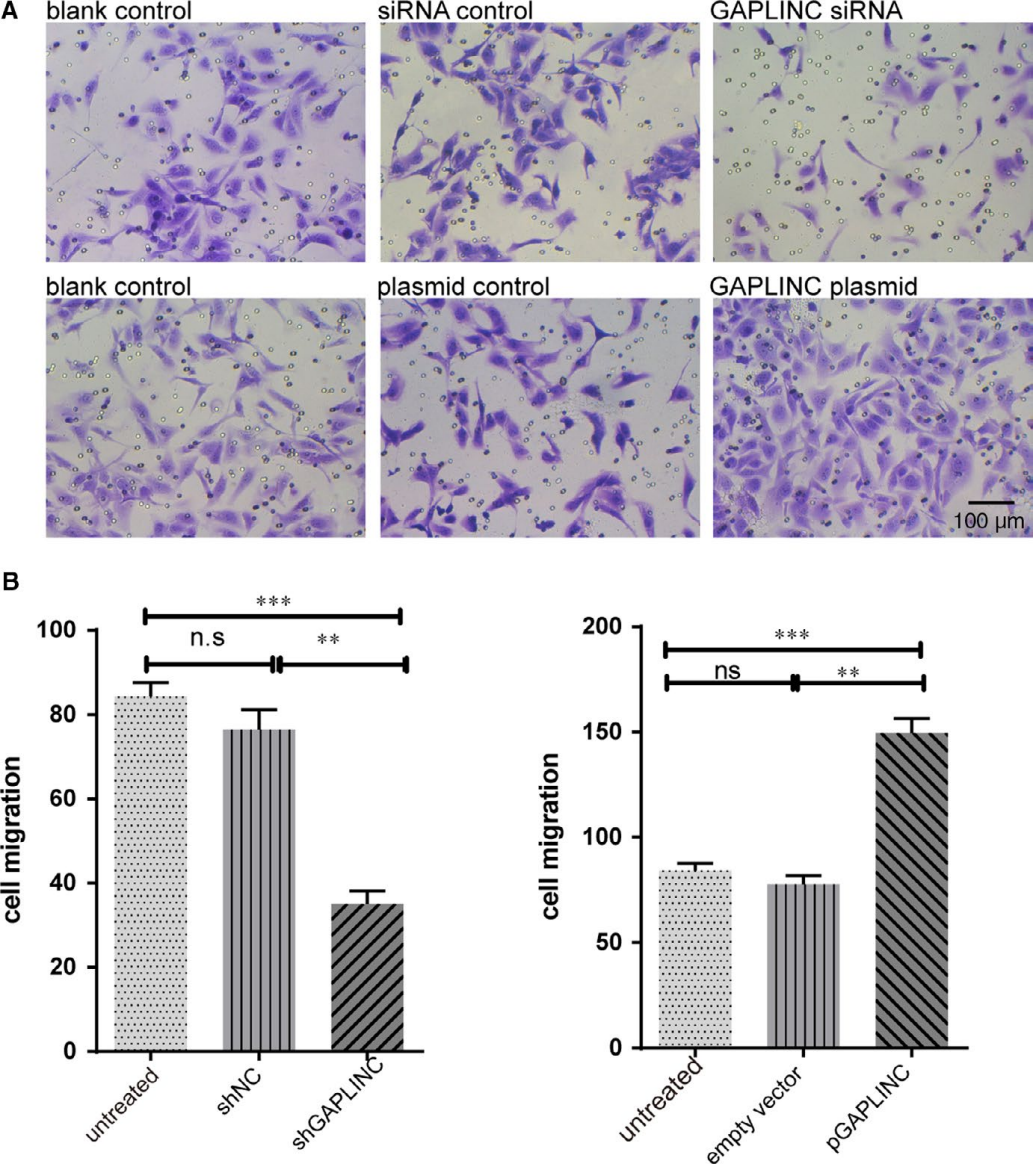
The influences of GAPLINC on cell migration. The cell was treated with siRNA control, GAPLINC siRNA, empty plasmid and GAPLINC plasmids. A, The representative figures of cell migration in different condition as labelled on top of each figure B. The summary cell migration in both GAPLINC knockdown (Left) and overexpression condition (right). The results are presented as mean ± SD for three independent experiments. Each experiment performed in triplicate. *: *P* < .05, **: *P* < .01, ***: *P* < .001, ****: *P* < .0001

Endothelial cell migration involved in many physiological processes including vascular repair and oxygen supply to tissues.^22^, ^23^ We then analysed the effects of GAPLINC on cell migration ability based on functional changes after knockdown or overexpression of GAPLINC conditions. The results revealed that knockdown of GAPLINC significantly attenuated cell mobility, and this effect was abolished by GAPLINC overexpression (Figure 4B). The representative data are shown in Figure 4A. This result is consistent with the findings from previous investigations on the cancer field, suggesting that overexpression of GAPLINC would influence local angiogenesis.^14^, ^15^, ^16^, ^17^

### 3.5 Enhanced GAPLINC expression accelerated vessel formation

**Figure 5:**
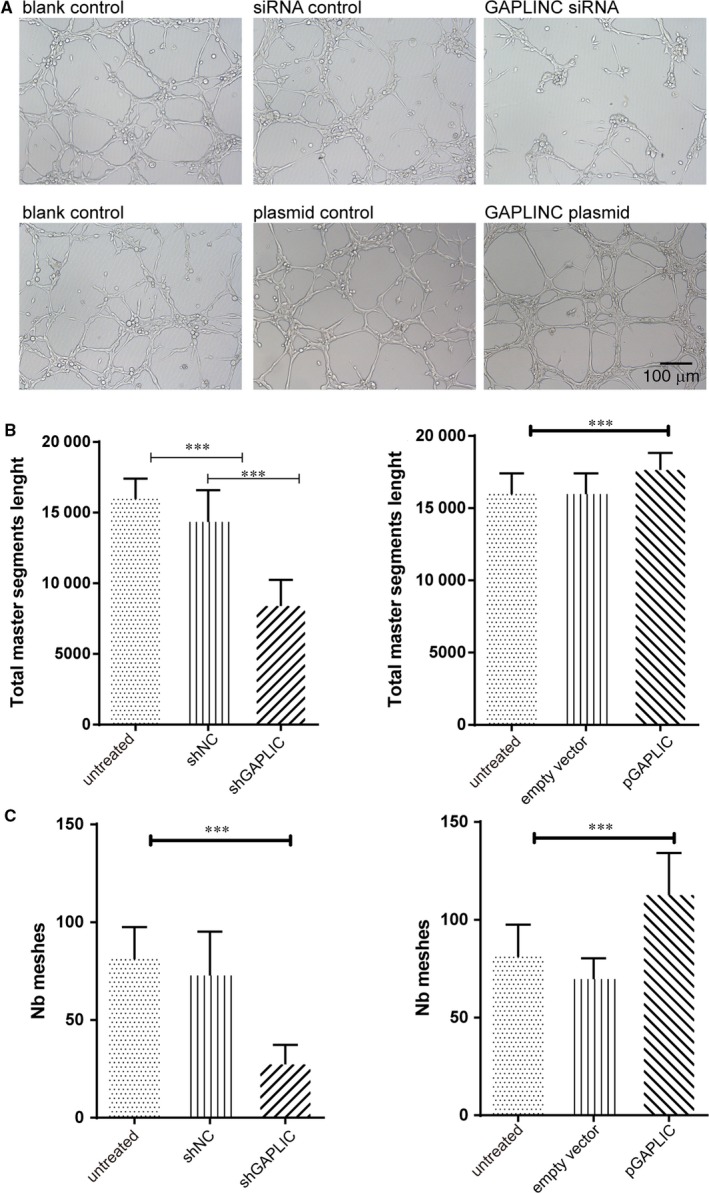
Enhanced GAPLINC expression accelerated vessel formation. A, The representative figures of vessel formation in different condition as labelled on top of each figure B. The summary of total master segments length measures the vessel formation in both GAPLINC knockdown (Left) and overexpression condition(right). C, The summary of the number of meshes measures the vessel formation in both GAPLINC knockdown (left) and overexpression condition(right). The results are presented as mean ± SD for three independent experiments. Each experiment performed in triplicate. *: *P* < .05, **: *P* < .01, ***: *P* < .001, ****: *P* < .0001

The formation of new blood vessels is a multi‐step process that ensures sufficient nutrient and oxygen supply. One of the key steps of this process is the vessel formation.^24^ Hence, we searched for the effects of GAPLINC expression on vessel formation including the total master segment length and the number of meshes (Nb meshes). We found the knockdown of GAPLINC significantly decreased the total master segment length and Nb meshes, whereas GAPLINC overexpression increased the total master segment length and Nb meshes as shown in Figure 5A‐C.

### 3.6 GAPLINC affected cell migration and vessel formation in hypoxia condition

**Figure 6:**
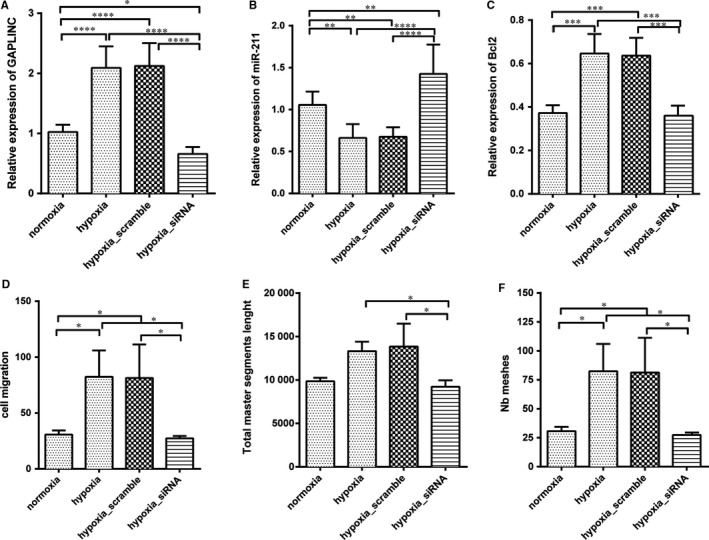
Silencing of GAPLINC under hypoxia condition reverse angiogenesis effects. The cell was treated with normoxia, hypoxia, hypoxia with scramble siRNA and hypoxia with GAPLINC siRNA, respectively. A, Expression level of GAPLINC quantified by qPCR. B, Relative expression level of miR‐211 quantified by qPCR. C, Relative expression level of Bcl2 quantified by Western blotting. D, The cell migration assays were performed using transwell chamber. E, Total master segment length. F, Total number of meshes. The results are presented as mean ± SD for three independent experiments. Each experiment performed in triplicate. *: *P* < .05, **: *P* < .01, ***: *P* < .001, ****: *P* < .0001

To further determine the relationship between GALINC induction and hypoxia, we performed a rescue experiment. Cells were treated under hypoxia with or without GAPLINC shRNA. The result demonstrated that the expression of GAPLINC was significantly increased under hypoxic condition, while silencing of GAPLINC decreased its expression level to normoxia condition. This indicates that hypoxia directly enhances GAPLINC expression (Figure 6A). Thereafter, we tested the expression of downstream molecules of mir‐211 and Bcl2. Data analysis showed that mir‐211 and Bcl‐2 were modulated by GAPLINC, and silencing of GAPLINC significantly increased the expression of miR‐211 but down‐regulated the expression of Bcl2 (Figure 6B and C). Moreover, the changes in cell migration and vessel formation were investigated. It was found that down‐regulation of GAPLINC under hypoxia condition significantly reduced cell migration as well as vessel formation, implying that the functional changes in cells were due to the effects of GAPLINC (Figure 6D and E). Taken together, these results indicated that GAPLINC was enhanced by hypoxia.

### 3.7 GAPLINC increased the expression of VEGFR and Dll4

**Figure 7:**
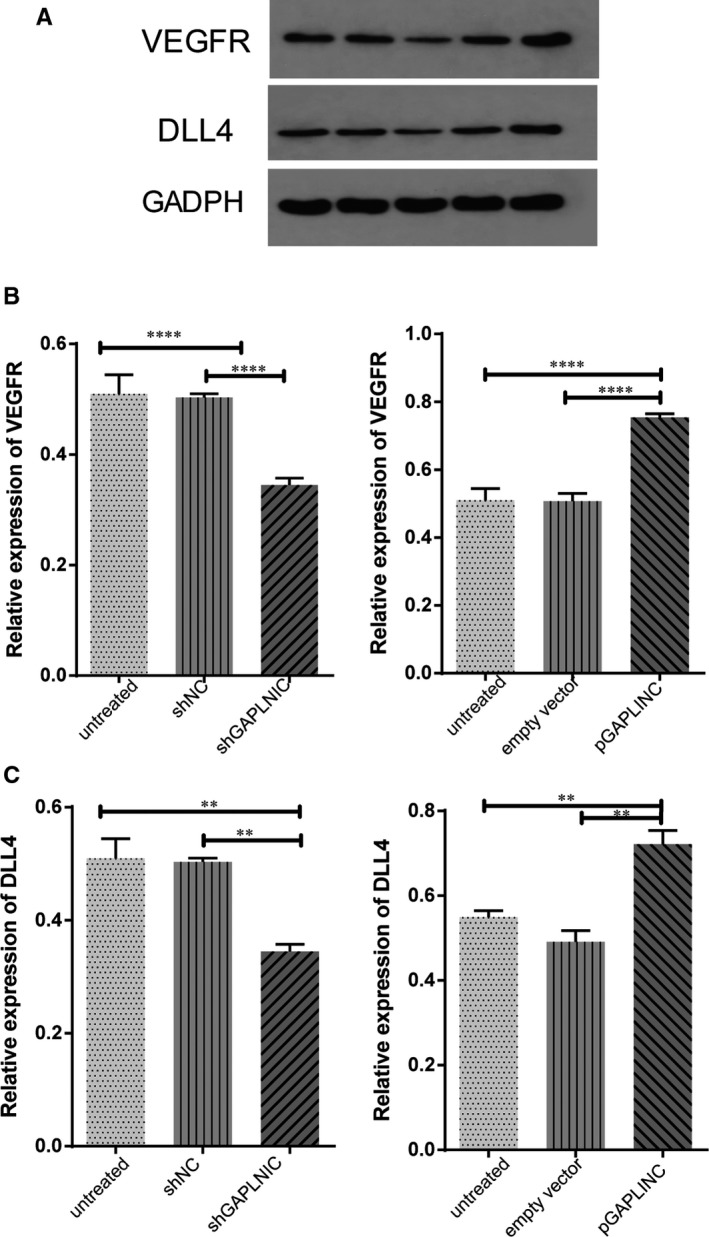
Effects of the expression level of GAPLINC on VEGFR and DLL4 expression in HUVECs. The treatment condition as indicated. Cells were lysed and proteins were transferred to PVDF membrane and stained with antibodies. A, Representative Western blot of VEGFR and DLL4 was shown. B, The summary of expression of VEGFR and DLL4 in different condition were shown. The results are presented as mean ± SD for three independent experiments. Each experiment performed in triplicate. *: *P* < .05, **: *P* < .01, ***: *P* < .001, ****: *P* < .0001

Since the GAPLINC can influence the cell migration and vessel formation in HUVEC cells, we explored the possibility that DLL4 and VEGFR pathway may involved in these phenotypes. It is well established that VEGFR and DLL4 are important factors for angiogenesis. DLL4 acting through Notch1/4 plays key roles such as regulating endothelial cells during normal and tumour angiogenesis. Thus, we investigated the effects of GAPLINC on DLL4 and VEGFR expression in HUVEC cells. The Western blotting assay showed that the protein levels of both DLL4 and VEGFR were increased significantly following GAPLINC overexpression, but these effects were reversed after GAPLINC knockdown settings, which pointed to the possibility that GAPLINC can modulate the VEGFR and DLL4 expression (Figure 7A‐C).

### 3.8 GAPLINC promotes vessel formation and migration by regulating MAPK and NF‐kB signalling pathways

We then attempted to determine the molecular mechanisms involved in the regulation of cell migration and vessel formation. Previous studies have found that MAPK and NF‐kB signalling pathways are involved in the regulation of angiogenesis.^25^, ^26^ Thus, we tested whether these two pathways play a role in angiogenesis in these study settings. We found that GAPLINC overexpression increased the level of phosphorylated p38, p65 and JNK, which are key targets of MAPK and NF‐kB signalling pathways, respectively. Silencing of GAPLINC showed opposite effects (Figure S1A‐B) suggesting that overexpression of GAPLINC activated the MAPK and NF‐kB signalling pathway in HUVEC cells.

## 4 DISCUSSION

Critical limb ischaemia is a clinical syndrome of ischaemic pain, rest pain, ischaemic ulceration and gangrene with a prevalence rate of about 1% in people aged 60‐90 years. Despite the advancements made towards developing therapies for CLI, about 25% of patients undergo limb amputations each year. Studies have shown that patients with diabetes have an approximate 4‐fold high risk of CLI.^27^ Non‐coding RNA such as RNA interference and lncRNAs have been demonstrated to have abilities to either up‐regulate or down‐regulate gene expression, implying that they can affect the disease progression or have therapeutic value.^28^ In this study, the expression of GAPLINC was increased under low oxygen condition, but glucose levels did not influence the expression of GAPLINC. Furthermore, we found that GAPLINC inhibited the anti‐apoptotic factor Bcl‐2 through miR‐211. Functional analyses showed that overexpression of GAPLINC increased cell migration and vessel formation which were attributed to the increased expression of VEGFR and DII4 receptors, effects that positively regulate CLI disease. Finally, we demonstrated that NF‐kB and MAPK signalling pathways regulated angiogenesis in HUVEC cells by modulating GAPLINC expression.

Recent studies have reported that lncRNAs are involved in several types of cancer including promoting cell proliferation and migration.^29^ However, it has been recognized that lncRNAs participate in diverse cellular processes such as chromatin remodelling, transcription, post‐transcriptional processing and intracellular trafficking.^30^, ^31^, ^32^ In this study, we investigated the role of GAPLINC in HUVEC cells and the potential role of lncRNA for CLI patient. GAPLINC was identified in human gastric cancer patients by global microarray.^14^ Moreover, it has been reported to be involved in several functions based on many tumour studies.^15^, ^18^ Here, we showed that the expression of GAPLINC was increased under low hypoxia condition and that high GAPLINC expression was associated with increased cell migration and vessel formation. The data also showed that silencing GAPLINC increased the expression of miR‐211 suggesting that GAPLINC can serve as a microRNA decoy. These findings also reveal that GAPLINC can modulate miR‐211 activities and can be utilized for disease treatment. miRNAs are small non‐coding RNAs which play many roles such as cell proliferation, cell differentiation and metabolism.^33^ In addition, previous studies reported that GAPLINC may regulate the expression of CD44 in HUVEC cells.^34^ It might also change during the manipulation of GALPINC. However, this study focused on determining the role of GALPINC on angiogenesis. Many studies have reported the interaction between miRNAs and lncRNAs. A previous study predicted that GAPLINC can interact with at least 10 different microRNAs, and among this, mir‐211 and mir‐34a were the top candidates. Nevertheless, this study did not test whether mir‐34a could also be potentially regulated by GAPLINC under low oxygen condition.^16^

Angiogenesis is the process that leads to the formation of new vascular networks. Several cellular signalling pathways can regulate this process, such as VEGF and its receptor (VEGFR), angiopoietin/Tie and Notch pathway.^35^ We showed that GAPLINC overexpression can increase cell migration and also vein formation which was attributed to the increased expression of VEGFR and DLL4 on the cell surface. In addition, we showed that GAPLINC overexpression activates the MAPK kinase and NF‐kB pathway. However, in this study, we did not investigate the interaction between GAPLINC with VEFGR and DLL4, as well as their signalling pathways. Nevertheless, our data revealed that GAPLINC overexpression can trigger similar biological behaviours like many cancer cells. Therefore, possible side‐effects of GAPLINC overexpression should be considered and how to regulate the expression of GAPLINC are critical for future directions. In this study, adenoviral vectors were used as delivery vector because it was well‐established vector including well‐defined biology, genetic stability, high transduction efficiency and less immunogenicity features.^36^, ^37^ In addition, recent years great success has been made for gene regulation system for gene therapy including tetracycline‐regulated system,^38^ rapamycin‐regulated system,^39^ RU486‐regulated systems^40^ and hypoxia‐regulated systems^41^ to control the level of expression or silencing of therapeutic genes in order to provide a balance between therapeutic efficacy and non‐specific toxicity. Thus, those regulation systems should be considered to include in our future design of vector and test the efficacy in animal models.

In summary, we explored the biological function of GAPLINC and its underlying mechanism in HUVECs. The results of this study indicate that GAPLINC was up‐regulated in hypoxia condition, and it was involved in the migration and invasion of HUVEC cells by regulating the miR‐211/Bcl2 signal pathway. Additionally, the expression of VEFGR and DLL4 was increased by the activation of MAPK and NF‐kB pathways. Researchers have successfully developed a tissue‐specific hypoxia‐responsive promoter to induce gene expression under hypoxia condition by using adeno‐associated vectors,^42^ and more and more lncRNAs have been found and tested in different diseases condition for their potential diagnostic and therapeutic roles.^43^ More interestingly, a recent study established VEGF‐expressing vector can increase angiogenesis which was very related to our study of GAPLINC^44^ implying that the possible of apply lncRNA for gene therapy. Taken together, these findings suggest that GAPLINC can be regarded as a novel therapy for CLI.

## CONFLICT OF INTEREST

There are no conflicts of interest.

## AUTHORS CONTRIBUTIONS

Yangyan He and Ziheng Wu: performed the research. Yangyan He, Hongkun Zhang and Donglin Li: designed the research study. Ziheng Wu, Chenyang Qiu, Hongkun Zhang and Donglin Li: contributed essential reagents or tools. Yangyan He, Xiaohui Wang, Yilang Xiang, Lu Tian, Yunjun He, Tao Shang, Qianqian Zhu, Xun Wang and Qinglong Zeng: collected the data. Xiaohui Wang and Yilang Xiang: analysed the data. Yangyan He: wrote the paper.

## Data Availability

All authors agreed to share data of this article according to Wiley's Data Sharing Policies.
