# Corrigendum

**DOI:** 10.1111/jcmm.15320

**Published:** 2020-06-14

In the article,^1^ the authors have reported 5 corrections as follows:

1. The legend of Figure 3 on page 8094, ‘F, Relative expression of miR‐211 when overexpressing GAPLINC expression’ should read as ‘F, Relative expression of Bcl‐2 when overexpressing GAPLINC expression’.
2. The results of 3.6 on page 8096, ‘To further determine the relationship between GALINC induction and hypoxia, we performed a rescue experiment’ should read as ‘To further determine the relationship between GAPLINC induction and hypoxia, we performed a rescue experiment’.
3. The results of 3.6 on page 8097, ‘Data analysis showed that mir‐211 and Bcl‐2 were modulated by GAPLINC, and silencing of GAPLINC significantly increased the expression of miR‐211 but down‐regulated the expression of Bcl2 (Figure 6B and C).’ should read as ‘Data analysis showed that miR‐211 and Bcl‐2 were modulated by GAPLINC, and silencing of GAPLINC significantly increased the expression of miR‐211 but down‐regulated the expression of Bcl2 (Figure 6B and C).’.
4. The headline of 3.7 on page 8097, ‘GAPLINC increased the expression of VEGFR and Dll4’ should read as ‘GAPLINC increased the expression of VEGFR and DLL4’.
5. The legend of Figure 7 on page 8098, ‘C, The summary of expression of DLL4 in different condition were shown.’ was missed out and should have been added.
6. The discussion on page 8098, ‘Functional analyses showed that overexpression of GAPLINC increased cell migration and vessel formation which were attributed to the increased expression of VEGFR and DII4 receptors, effects that positively regulate CLI disease.’ should read as ‘Functional analyses showed that overexpression of GAPLINC increased cell migration and vessel formation which were attributed to the increased expression of VEGFR and DLL4 receptors, effects that positively regulate CLI disease.’.
