# Supplementary figures and images for: Long non‐coding RNA GAPLINC promotes angiogenesis by regulating miR‐211 under hypoxia in human umbilical vein endothelial cells
Source: J Cell Mol Med. 2019 Oct 7;23(12):8090–100. doi: 10.1111/jcmm.14678 (PMC6850972; doi:10.1111/jcmm.14678)

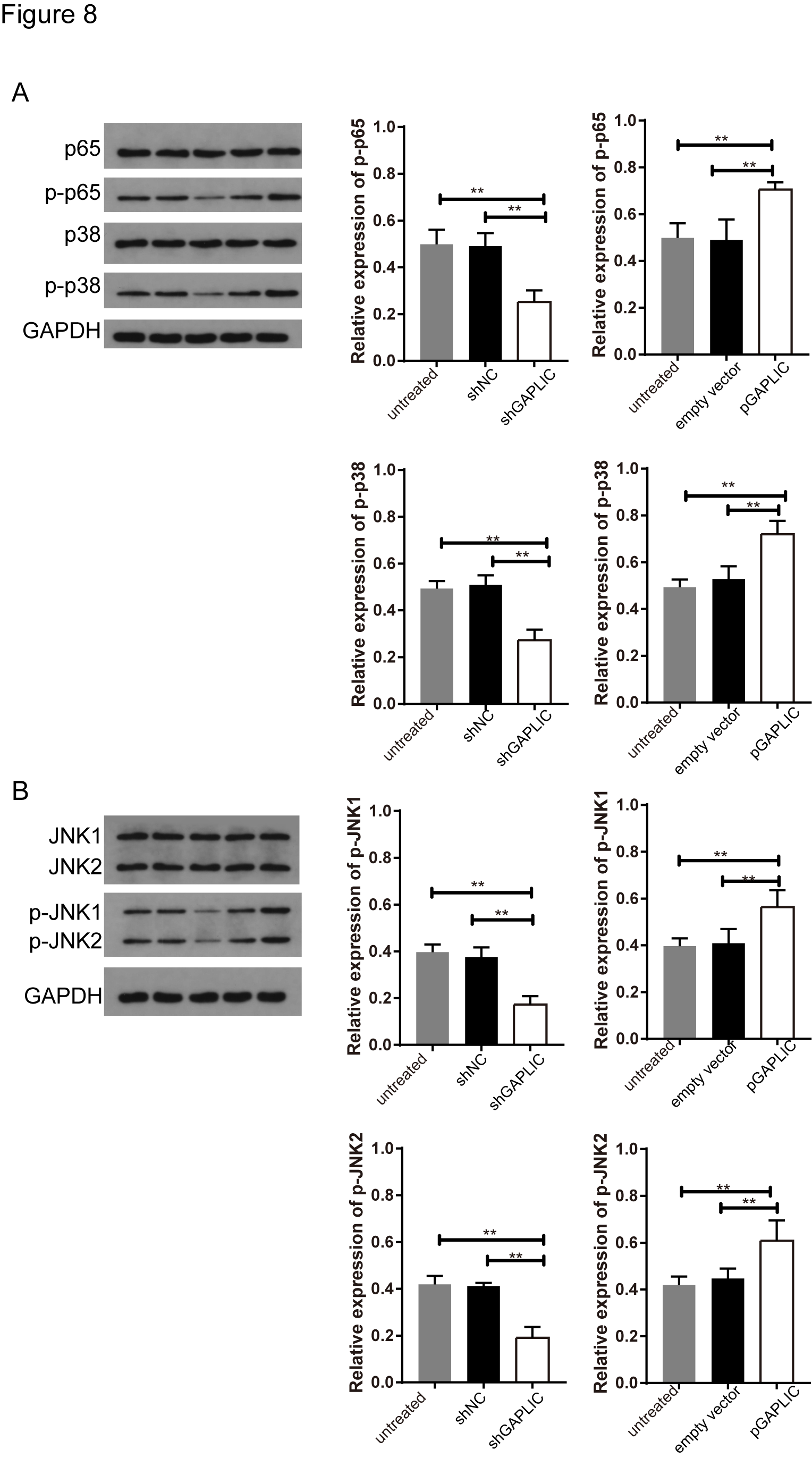

Supplement: Supplementary file 1 [file JCMM-23-8090-s001.tif]
